# Zirconium Phosphonate Sorbent Materials—Synthesis, Characterization, and Application for Copper Removal from Acidic Wastewater

**DOI:** 10.3390/ma18102333

**Published:** 2025-05-16

**Authors:** Marta Marszałek, Marcin Piotrowski, Bożena Druzgała, Zbigniew Wzorek

**Affiliations:** Cracow University of Technology, Faculty of Chemical Engineering and Technology, Warszawska 24, 31-155 Krakow, Poland

**Keywords:** zirconium phosphonate, aminotris(methylenephosphonic acid), copper sorption, chelating resin, acidic wastewater

## Abstract

Copper removal from wastewater is a major challenge in the treatment of both hydrometallurgical copper recycling effluents and mining wastewater. The use of sorbents is considered the most efficient and environmentally friendly method for this purpose. Zirconium aminotris(methylenephosphonates) exhibit excellent sorption properties towards many metal ions. Therefore, the goal of this research was to synthesize amorphous zirconium phosphonate materials with a wide range of P:Zr molar ratios (0.5–100) in the reaction mixture and under mild conditions. The obtained sorbents were characterized using ATR-FTIR, XRD, SEM-EDS, CHN analysis, and pH titration. The sorption properties of the zirconium aminotris(methylenephosphonates) were studied in pure copper solutions and in the complex acidic solutions (pH 1.6–3.2). The results were compared with those for the commercially available resins designed to capture copper containing iminodiacetic acid and bispicolylamine as surface groups. Zirconium aminotris(methylenephosphonate) synthesized at a P:Zr molar ratio of 50:1 shows fast sorption kinetics and the best sorption properties. The maximum sorption capacity in pure copper solutions is 62.3 mg/g, and its affinity for copper ions is comparable to that of reference resins *(log K_d_* = 2.7–3.9). Moreover, this sorbent can be easily regenerated with 1 M solutions of HCl, HNO_3_, or H_2_SO_4_ (Cu^2+^ recovery up to 95%).

## 1. Introduction

Copper is a metal that is essential for modern living. It is widely used in both domestic and industrial applications. Due to its great economic importance, copper has been recognized as a strategic and critical raw material for the European Union [1]. Moreover, copper is recognized as a crucial energy transition material. According to an International Energy Agency report, global demand for copper will increase in the coming years from 5.4 million tons per year in 2021 to 16.3 million tons per year in 2040. The key driver for such high growth of copper demand is especially the cleantech sector [2].

Greater copper consumption will result in greater and more intensive exploitation and processing of copper deposits, as well as increased demand for copper recycling technologies. The role of so-called urban mining in meeting future copper demand is currently widely discussed [3,4]. Copper is relatively easy to recover from many products, such as wires and scraps of copper containing alloys [5]. The recovery process typically includes scrap collection, sorting and separation, and recovery of metal through a metallurgical process [6].

Nevertheless, there are many types of waste where chemical methods must be used to recover copper, e.g., end-of-life electronic products (so-called e-waste) [7,8] or spent lithium ion batteries [9]. It is important to mention that the content of copper in used printed circuit boards is 5–65% [7,10]. The global production of e-waste in 2014 was 40 million tons, and it increased to 62 million tons in 2023. Lithium ion batteries can contain up to 11% copper, which is used as current collectors and for wiring [9]. Moreover, recent studies indicate that recovery of copper from e-waste can significantly reduce its carbon footprint (by 71–97%) compared to its primary extraction [10].

In general, the more complex the device, the less cost-effective are mechanical methods based on disassembly of the device and sorting of materials (especially for miniaturized and compact devices). In such cases, chemical methods (especially hydrometallurgy and pyrometallurgy) are preferred, often combined with physical separation (screening, magnetic and gravity separation, flotation) and processing (crushing, milling) [7]. Pyrometallurgical methods used to recover metals from e-waste are considered to be highly energy-intensive (up to 2000−2500 kWh per ton of material) and therefore not very sustainable. Hydrometallurgical methods operate at low temperatures, and energy consumption is significantly lower (500−800 kWh per ton of material). However, on the other hand, hydrometallurgy consumes large amounts of water and generates hazardous wastewaters (often acidic) containing i.a. heavy metals [8]. To address this problem, many scientific studies [11,12,13,14] have been carried out in recent years.

Wastewater management is also a major challenge for the copper mining and processing industry [15], as this type of wastewater is generated in large quantities, often acidic and saline, has a high sulphate content, and contains heavy metals (Cu, Ni, Zn, Co, Mn, Fe, Al, As, and Cr) in relatively low but environmentally hazardous concentrations [16,17]. Direct discharge of untreated wastewater from the copper industry into the environment can lead to contamination of soils and ground and surface water [18]. Copper contamination of rivers is particularly dangerous, as it can lead to serious damage to aquatic biota [19]. At very low concentrations, copper is an essential element for any living organism (due to copper-containing proteins, which are necessary for cellular respiration, among other things), while at higher concentrations it causes serious diseases and even death [15,20]. Therefore, already in the 1990s, many countries introduced strict limits on copper content in drinking water (below 2 mg/L according to WHO recommendations) [21,22]. There are also limits on copper concentration in industrial wastewater discharged into the water or ground; for example, in Poland, it is 0.5 mg/L [23].

Removing copper to such low levels, especially from acidic wastewater, is still difficult and often requires the use of sophisticated methods [18,24]. Considering the cost effectiveness, simplicity of operation, environmental friendliness, high removal efficiency, and amount of secondary waste produced (during the process), sorption methods are most often considered the best solution for removing copper from industrial wastewater [15,18,24,25,26,27]. Due to the large volume of wastewater that needs to be processed, the preparation of inexpensive sorbents that have high metal-binding capacity is of considerable significance [15]. For this purpose, the following classes of materials were tested, including, among others, natural clay minerals [28], biochar [29], alginates [30], and amorphous metal(IV) phosphates [31,32,33]. The last mentioned class of materials can be used in a continuous process, as was proved for the TiO(OH)H_2_PO_4_·H_2_O using real acid mine drainage [31]. There are also commercially available resins designed to capture copper, such as polystyrene–divinylbenzene resins containing iminodiacetic acid, aminophosphonic acid, and bispicolylamine surface groups, but the prices of such materials are relatively high [34,35].

In our previous work, we showed that amorphous phosphates synthesized under mild conditions exhibit promising affinity for copper in acidic media (pH 2) and exceptionally fast sorption kinetics (in the best cases, equilibrium was reached in no more than 7 min) [33]. Zirconium phosphate synthesized at a high molar ratio of phosphoric acid to zirconium ions in the reaction mixture showed comparable performance to some commercial ion exchange resins, so we decided to explore this area further, looking for materials with higher selectivity towards copper ions. We assumed that the high sorption rate for the tested phosphates is associated with their amorphous nature. Therefore, we tried to synthesize novel materials with other ligands, still under mild conditions (to suppress the crystallization process). Literature reviews show that materials containing organophosphonate ligands exhibit promising sorption properties [36,37,38,39,40,41,42,43,44,45,46,47].

In order to develop a low-cost sorbent, the production of which can be easily scaled up, only inexpensive industrially produced chemicals should be sought for its preparation. Aminotris(methylenephosphonic acid) (ATMP) or nitrilotris(methylene)triphosphonic acid (preferred IUPAC name) was selected because it is produced on a large scale (in the European Union, according to the European Chemical Agency, the scale of production of this compound ranges from 10 kt to 100 kt [48]) and it is relatively cheap, non-toxic, and widely used in industry (as an antiscalant in closed water systems) [49]. Moreover, ATMP (with linear formula N[CH_2_P(O)(OH)_2_]_3_) contains aminophosphonic groups in its structure, which are used as copper-selective groups in commercially available resins [34], and this ligand can form branched cross-linked structures due to the similar reactivity of the three aminophosphonic groups in the molecule. Zirconium aminotris(methylenephosphonate) (Zr-ATMP) materials have been reported as promising sorbents with fast kinetics and high stability, even in strongly acidic media [41,42]. Numerous reports indicate that they are particularly effective in the sorption of heavy metals [44,45,46,47], alkaline earth metals [41], and lanthanides [36,42].

The aim of this research was to synthesize amorphous zirconium aminotris(methylenephosphonate) sorbents with a wide range of P:Zr molar ratios in the reaction mixture (0.5:1–100:1) and under mild (atmospheric pressure, room temperature) and easily scalable conditions, characterize their elemental composition and structure, and study their sorption properties for the removal of copper, especially from acidic solutions (similar to streams from copper mining). The sorption properties of Zr-ATMP materials were discussed in relation to selected state-of-the-art commercially available products dedicated to copper capture. Two chelating resins, AmberSep™ M4195 (Sigma-Aldrich, St. Louis, MO, USA) containing bispicolylamine groups and Puromet™ MTS9300 (Purolite, King of Prussia, PA, USA) containing iminodiacetic acid groups, were selected as reference materials. To the best of our knowledge, this is the first study that systematically examines amorphous Zr-ATMP-based materials synthesized under mild conditions and according to an easily scalable procedure used for the sorption of copper from acidic solutions (pH 1.6–3.2) and compares their properties with those of selected commercially available chelating resins.

This research is a continuation of our previous work [33], and the results obtained for zirconium phosphonate sorbents were discussed in relation to findings obtained for phosphate sorbents (titanium(IV), zirconium(IV), and cerium(IV) phosphates).

## 2. Materials and Methods

### 2.1. Materials

All chemicals used to synthesize zirconium phosphonate sorbent materials and to study their properties were of analytical grade. Nitrilotris(methylene)triphosphonic acid solution 50% in H_2_O was delivered by Sigma Aldrich (St. Louis, MO, USA). Zirconium carbonate basic hydrate was supplied by Alfa Aesar (Ward Hill, MA, USA). Nitric acid 65%, sulfuric acid 95%, hydrochloric acid 37%, potassium sulphate anhydrous, sodium sulphate anhydrous, magnesium sulphate anhydrous, calcium chloride anhydrous, manganese(II) sulphate monohydrate, cadmium nitrate tetrahydrate, copper(II) sulphate pentahydrate, nickel(II) nitrate hexahydrate, zinc sulphate heptahydrate, iron(III) nitrate nonahydrate, and sodium hydroxide were provided by Chempur (Piekary Śląskie, Poland).

Two commercially available chelating resins designed for selective copper removal were used as received. Puromet^TM^ MTS9300 iminodiacetic acid chelating resin for copper, cobalt, nickel, and zinc removal was obtained from Purolite (King of Prusia, PA, USA). AmberSep^TM^ M4195 bispicolylamine chelating resin for selective removal of copper, nickel, and cobalt from strong acid solutions was purchased from Sigma-Aldrich (St. Louis, MO, USA). Both resins are macroporous and produced on the basis of a copolymer of styrene and divinylbenzene [50,51]. According to the product data sheets of the resins, the copper capacity for Puromet^TM^ MTS9300 is 50 g/L [50], while for AmberSep^TM^ M4195 it is ≥35 g/L [51].

#### Solutions Employed in Sorption Studies

To determine the sorption properties of the obtained materials, the following solutions were used:

1. Nine solutions with different concentrations of Cu^2+^, i.e., 5 mg/L, 10 mg/L, 20 mg/L (designated as Solution 1), 50 mg/L, 100 mg/L, 200 mg/L, 500 mg/L, 1000 mg/L, and 2000 mg/L;
2. A solution designated as Solution 2 containing 20 mg/L Cu^2+^, 1 g/L Na^+^, and 1 g/L K^+^;
3. A solution designated as Solution 3 containing 20 mg/L Cu^2+^, 1 g/L Na^+^, 1 g/L K^+^, 200 mg/L Mg^2+^, and 20 mg/L Ca^2+^;
4. A solution designated as Solution 4 simulating acid wastewater from copper mining with the following composition: 20 mg/L Cu^2+^, 1 g/L Na^+^, 1 g/L K^+^, 200 mg/L Mg^2+^, 20 mg/L Ca^2+^, 20 mg/L Cd^2+^, 20 mg/L Mn^2+^, 20 mg/L Ni^2+^, 20 mg/L Zn^2+^, and 10 mg/L Fe^3+^.

The solutions were prepared by dissolving appropriate amounts of metal salts in deionized water.

### 2.2. Synthesis of Zirconium Phosphonate Sorbents

Eight zirconium aminotris(methylenephosphonates) (designated as Zr-ATPMP 1, Zr-ATMP 2, Zr-ATMP 3, Zr-ATMP 4, Zr-ATMP 5, Zr-ATMP 6, Zr-ATMP 7, and Zr-ATMP 8) were obtained through the precipitation method using a procedure similar to that described earlier [52]. All syntheses were carried out under mild conditions, i.e., at atmospheric pressure and room temperature.

The first step was to prepare a zirconium(IV) oxynitrate solution by reacting 33.3 g of zirconium carbonate basic hydrate with 35 mL of 65% HNO_3_. The resulting solution was then diluted with 400 mL of deionized water. Subsequently, a 50% ATMP solution was added drop by drop to the diluted solution of zirconium(IV) oxynitrate, under continuous stirring, in such an amount to achieve the assumed P:Zr molar ratio in the reaction mixture (Table 1). After completion of the precipitation, the obtained suspension was stirred for 1 h and then left to age for 24 h. Afterwards, the supernatant was decanted, and the remaining precipitate was rinsed with deionized water until the pH of the rinsing reached approximately 3.5. In the next step, the sorbent was centrifuged and air-dried in a laboratory oven at 80 °C for 24 h. Finally, the obtained white product was ground down.

### 2.3. Physicochemical Characterization

The crystal structure of the obtained materials was evaluated through powder X-ray diffraction (XRD) using a SmartLab SE powder X-ray diffractometer coupled with a HyPix 400 detector (Rigaku, Tokyo, Japan). XRD patterns were recorded in the range 2θ between 5° and 80° at the scan speed of 3°/min using a Cu radiation source operating at 40 kV and 50 mA.

To identify the functional groups present in the obtained sorbents, Fourier transform infrared spectroscopy (FTIR) was used. The analysis was conducted on a Nicolet iS5 FTIR spectrometer equipped with an iD7 attenuated total reflection (ATR) accessory with diamond crystal (Thermo Fisher Scientific, Waltham, MA, USA). FTIR spectra were recorded in transmittance mode in the 500–4000 cm^−1^ range, with 32 scans per sample.

Morphologies of zirconium phosphonates were observed using a high-resolution scanning electron microscope (SEM) Apreo 2 S LoVac (Thermo Fisher Scientific, Waltham, MA, USA) at an acceleration voltage of 2 kV and magnification of 50,000×. Elemental analysis of materials was carried out through energy dispersive X-ray spectroscopy (EDS). EDS point microanalyses were performed using the UltraDry X-ray detector (Thermo Fisher Scientific, Waltham, MA, USA) at three points on the sample surface (the results were averaged). For SEM-EDS analysis, a dry powder sample was attached to an aluminum sample holder with conductive double-sided carbon tape. The excess of the sample was removed.

To determine the content of carbon, hydrogen, and nitrogen in the Zr-ATMP sorbents, elemental analysis in CHN mode (CHN analysis) was performed using the Perkin Elmer 2400 Series II CHN Elemental Analyzer (PerkinElmer, Waltham, MA, USA). Samples were combusted at 950 °C.

The residue on ignition (ROI) was determined gravimetrically. The sample was placed in a crucible and calcined in a muffle furnace at 950 °C for 4 h. The resulting inorganic residue was weighed. Calcination was repeated to ensure that a constant mass was achieved.

Low-temperature nitrogen sorption was recorded on a Micromeritics ASAP 2020 (Micromeritics, Norcross, GA, USA) analyzer at 77 K. Samples were degassed under vacuum at 150 °C for 24 h. The specific surface area was determined using the Brunauer–Emmett–Teller method (BET) in the pressure range of p/p_0_ = 0.05–0.18. The size of the pores was determined using the Barrett–Joyner–Halenda method (BJH).

Total ion exchange capacity was evaluated through direct titration of sorbent samples using a sodium hydroxide solution. A 0.1 g portion of each material was dispersed in 80 mL of deionized water and placed in a thermostated vessel equipped with a magnetic stirrer. Titration was conducted at 20 ± 0.2 °C using 0.1 M NaOH solution. The titrant was continuously dosed using a syringe pump with a flow rate of 1 mL/h (ca. 1 meq/g_sorbent_/h). The pH of the suspension was recorded using the multifunction meter CX-701 (ELMETRON, Zabrze, Poland) after every 5 min. Based on the pH values measured before and after ion exchange, the Na^+^ uptake (expressed in meq/g) was calculated. Titration was carried out until the pH of the solution reached a steady value or until the sorbent was completely dissolved.

The concentration of zirconium in samples after synthesis (precipitation), sorption, and regeneration, as well as the concentration of phosphorus in samples after sorption and regeneration, were determined through inductively coupled plasma mass spectrometry (ICP-MS) on Elan DRC-e (PerkinElmer Inc., Waltham, MA, USA). The analysis was carried out with an acquisition time for a single element of 1500 ms and a flushing time between samples of 2 min. The calibration curve for zirconium was prepared in the range of 1–10 μg/L and for phosphorus in the range of 10–100 μg/L.

### 2.4. Sorption Studies

All of the sorption experiments were conducted at room temperature using the batch contact method. The sorption tests for Solution 2, Solution 3, and Solution 4 were carried out at a pH of approximately 2. The pH of a given solution was corrected with a 12% H_2_SO_4_ solution.

To study sorption isotherms and kinetics, 0.2 g of Zr-ATMP sorbent or chelating resin was added to 50 mL of solution containing different concentrations of copper ions (5 mg/L, 10 mg/L, 20 mg/L, 50 mg/L, 100 mg/L, 200 mg/L, 500 mg/L, 1000 mg/L, and 2000 mg/L) or a solution similar to acid wastewater from copper mining while stirring continuously. After contact times of 1 min, 5 min, 15 min, 30 min, 60 min, and 24 h, a sample of the mixture was taken, and the solid was immediately separated from the solution through centrifugation. The concentration of metal ions in the initial solution and the supernatant was determined through atomic absorption spectrometry (AAS) using a Perkin Elmer 1100B atomic absorption spectrometer (PerkinElmer, Waltham, MA, USA). Details of the method were described in our previous report [33]. Kinetic and isotherm tests were performed in duplicate, and the results were averaged.

The amount of metal ions adsorbed at a specified time was calculated using Equation (1):

(1) $$q_{t}=\frac{\left(C_{0}-C_{t}\right)\cdot V}{m}$$

where *C*_0_ (mg/L) and *C_t_* (mg/L) are the initial metal ion concentration and the concentration at a specified time, respectively, *V* (L) is the volume of the solution, *m* (g) is the mass of the Zr-ATMP sorbent or the chelating resin, and *q_t_* (mg/g) is the sorption capacity at a specified time.

The amount of metal ions adsorbed at equilibrium, i.e., after 24 h of contact of the Zr-ATMP sorbent or the chelating resin with the solution, was calculated based on Equation (2):

(2) $$q_{e}=\frac{\left(C_{0}-C_{e}\right)\cdot V}{m}$$

where *C_e_* (mg/L) is the metal ion concentration at equilibrium and *q_e_* (mg/g) is the sorption capacity at equilibrium.

To investigate the interaction between the surface of Zr-ATMP sorbents and copper ions, the Langmuir and the Freundlich isotherm models were used. The non-linear form of the Langmuir isotherm model is represented by Equation (3):

(3) $$q_{e}=\frac{q_{m}K_{L}C_{e}}{1+K_{L}C_{e}}$$

where *q_m_* (mg/g) is the maximum sorption capacity and *K_L_* (L/mg) is the Langmuir isotherm constant. The non-linear form of the Freundlich isotherm model is expressed by Equation (4):

(4) $$q_{e}=K_{F}C_{e}^{\frac{1}{n}}$$

where *K_F_* ((mg/g)·(L/mg)^1/*n*^) is the Freundlich isotherm constant and *n* (dimensionless) is the heterogeneity factor.

To study the affinity and selectivity of the obtained Zr-ATMP materials to copper ions, 0.2 g of selected sorbent was added to 50 mL of Solution 1, Solution 2, Solution 3, or Solution 4 and stirred vigorously. After 24 h of contact, the sorbent was separated from the solution through centrifugation. The concentration of metal ions before and after sorption was determined through the AAS method. The competitive adsorption experiment was repeated three times, and the average value was taken as the result.

To assess the affinity of zirconium phosphonate sorbents for copper ions, the distribution coefficients *K_d_* (mL/g) were calculated using Equation (5):

(5) $$K_{d}=\frac{C_{0}-C_{e}}{C_{e}}\cdot \frac{V}{m}$$

To evaluate the selectivity of zirconium phosphonate materials towards copper ions, the separation factors were determined according to Equation (6):

(6) $${SF}_{Cu/Me}=\frac{K_{d(Cu)}}{K_{d(Me)}}$$

where *K_d_*_(*Cu*)_ (mL/g) and *K_d_*_(*Me*)_ (mL/g) are the distribution coefficient of copper ions and the distribution coefficient of selected metal ions, respectively.

### 2.5. Studies on the Effect of pH on the Sorption Properties of Zr-ATMP 7 Sorbent

To evaluate the effect of the pH of a solution simulating acid wastewater from copper mining on the effectiveness of the best-performing zirconium aminotris(methylenephosphonate) sorbent, tests were conducted in the pH range of 1.6–3.2. To start the experiment, 0.2 g of Zr-ATMP 7 sorbent and 50 mL of Solution 4 were placed in a thermostated vessel equipped with a magnetic stirrer and a pH sensor. The pH was adjusted to the expected value with a 2 M HCl solution dosed with a syringe pump. After 24 h of contact of the sorbent with Solution 4, a sample of the mixture was taken and centrifuged, and the supernatant was analyzed through the AAS method. After sampling, the pH of the mixture was adjusted to the next selected value.

### 2.6. Regeneration Studies

To assess the reusability of Zr-ATMP 7 sorbent, a desorption experiment was conducted. After sorption equilibrium was reached, i.e., after 24 h of contact with solution S4, the Zr-ATMP 7 sorbent was separated through centrifugation. The supernatant was discarded (a sample of solution was taken and analyzed using the AAS method), and the solid was washed and centrifugated with three aliquots (10 mL each) of deionized water (samples of washing water were also analyzed through the AAS method). The washed sorbent was regenerated by contacting it for 24 h with two portions (3 mL each) of a suitable acid solution. Three acids were used (HCl, HNO_3_, and H_2_SO_4_) at three different concentrations (0.1 M, 0.5 M, and 1.0 M). The recovery of the selected metal ions (%) was calculated according to Equation (7):

(7) $$Recovery=\frac{C_{reg}\cdot V_{reg}}{q_{e}\cdot m}\cdot 100\%$$

where *C_reg_* (mg/L) is the concentration of the selected metal ions in the regenerated solution, *V_reg_* (L) is the volume of the regeneration solution, *q_e_* (mg/g) is the equilibrium capacity for the selected metal ions determined during sorption, and *m* (g) is the mass of the Zr-ATMP 7 sorbent.

## 3. Results and Discussion

### 3.1. Synthesis and Characterization of Zirconium Aminotris(methylenephosphonate) Sorbents

There are several reports in the literature concerning the synthesis of zirconium aminotris(methylenephosphonate) sorbents [36,42,43,45,46,47]. However, data on the effect of the ratio of aminotris(methylenephosphonic) acid to zirconium ions on the properties of the prepared materials are scarce. Moreover, typically, zirconium(IV) propoxide [36,43] or zirconyl chloride [42,46,47] is used as a source of zirconium ions; in our study, we applied zirconium oxynitrate. In the case of zirconium, the nitrate ligand is less coordinating than the chloride, which can affect the structure of the sorbent. As in many other cases involving inorganic sorbents, the more common method of synthesis is the hydrothermal method, which requires the use of high temperatures and pressures [36,42,43,46]. Therefore, in this work, the synthesis of Zr-ATMP materials was conducted under mild conditions, which should greatly simplify process scaling.

**Figure 1:**
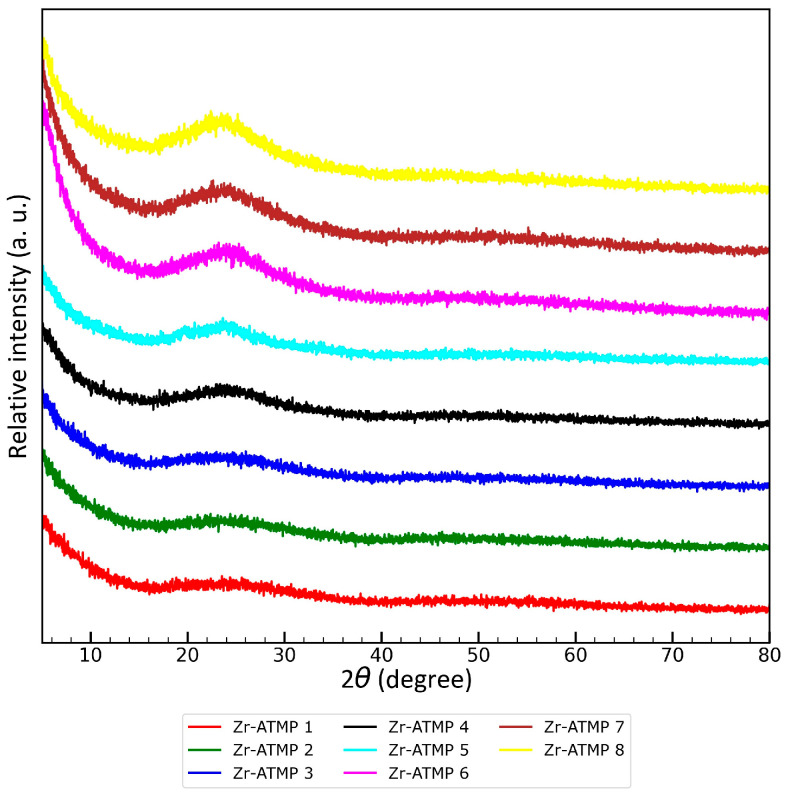
XRD patterns of Zr-ATMP sorbents.

The X-ray diffraction patterns of the obtained Zr-ATMP sorbents are presented in Figure 1. All materials are fully amorphous. The recorded diffraction patterns are virtually identical to a typical amorphous halo in the range of 20 to 30° (with a maximum at 24°). Precipitation of the metal(IV) phosphonate at low temperature favors (as anticipated) the formation of amorphous materials, so our observations are consistent with general trends observed in the literature.

Usually, the molar ratio of P:Metal(IV) in the reaction mixture is the key parameter determining the structure and the properties of the metal(IV) phosphate/phosphonate sorbent. To assess the impact of this parameter on the performance properties of the sorbents, syntheses were performed using different P:Zr(IV) molar ratios (0.5, 1, 2, 5, 10, 25, 50, 100). In our previous work [33] on metal(IV) phosphates, we showed that high P:Metal(IV) molar ratios have a positive effect on sorption properties. Therefore, in the present study, the range of tested P:Zr(IV) molar ratios was widened.

**Figure 2:**
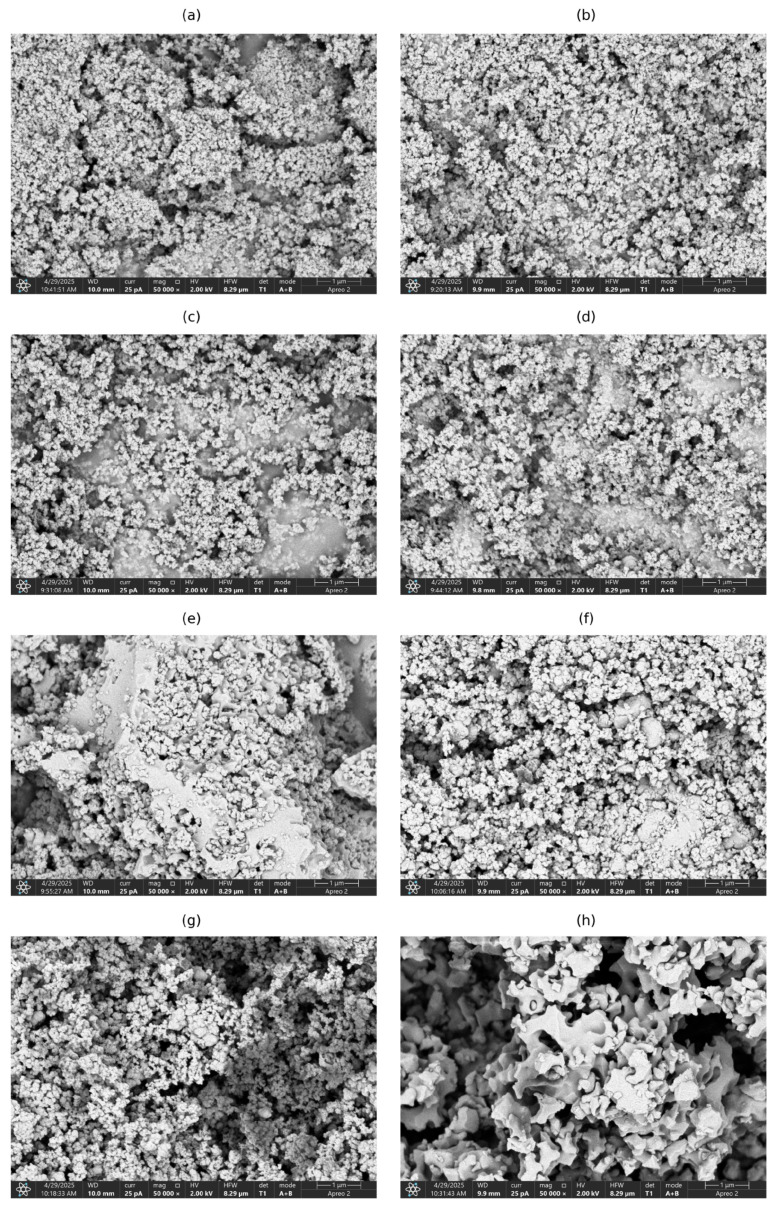
SEM images of (**a**) Zr-ATMP 1, (**b**) Zr-ATMP 2, (**c**) Zr-ATMP 3, (**d**) Zr-ATMP 4, (**e**) Zr-ATMP 5, (**f**) Zr-ATMP 6, (**g**) Zr-ATMP 7, and (**h**) Zr-ATMP 8 recorded at magnification 50,000×.

The surface morphology of sorbents was characterized using a scanning electron microscope. Figure 2 shows SEM images at magnification of 50,000×. Samples synthesized at low P:Zr(IV) molar ratios consist of round-shaped nanoparticles clumped together. The sizes of such particles are smaller than at least 100 nm. The particle size distribution is rather small. The images for sorbents from Zr-ATMP 1 to Zr-ATMP 4 are very similar.

It is likely that the observed particles have an internal structure and that they are porous, as confirmed by images recorded at higher magnification (these images were not included due to poor quality).

Zr-ATMP 5 has a significantly different morphology—larger irregular clumps of material (size of approximately 2–10 μm) are covered by smaller, also irregular fragments. This appears to be the result of coalescence of the smaller particles. A closer look at the image reveals round shapes of voids in the material (probably macropores). The morphology of Zr-ATMP 6 and Zr-ATMP 7 samples is intermediate between Zr-ATMP 5 and sorbents synthesized at low P:Zr(IV) molar ratio. Some larger irregular particles are present, as well as round-shaped nanoparticles with a wrinkled surface. In this case, the round-shaped nanoparticles are slightly larger than in sorbent samples from Zr-ATMP 1 to Zr-ATMP 4.

Zr-ATMP 8 has a completely different morphology than the other sorbents. The image is most similar to the Zr-ATMP 5 sample, but, in this case, there are no smaller particles. The material consists of a foam-like framework. There are clearly visible macropores (sizes of about 0.1–1 μm). The material that builds the cell walls of such a foam consists of fully fused smaller nanoparticles. A closer look at the image reveals poorly visible boundaries between such particles (cracks).

To sum up, the P:Zr(IV) molar ratio used in the synthesis also has a significant effect on the morphology of the materials obtained. It seems that a higher ATMP to Zr(IV) molar ratio favors the coalescence of particles produced during nucleation, which is directly associated with the precipitation rate. It is likely that better control of hydrodynamics during the process will also effect the morphology of the obtained material, which opens the possibility for further optimization.

The composition of the obtained materials was studied using SEM-EDS and elemental (CHN) analysis. In addition, the residue on ignition at 950 °C was determined.

The results of SEM-EDS analyses are presented in Table 2 and Table 3. Moreover, the composition ranges at different points, as well as the ratio of the range to the mean (the relative range), were calculated. The results presented should be considered semi-quantitative (trends rather than absolute values should be interpreted), as powder samples were analyzed. According to the literature, it is known that one of the assumptions of EDS analyses is the flatness of the studied sample. Results for powder may be burdened with greater uncertainties [53].

Because precipitation of Zr-ATMP sorbents is a rapid process (it occurs almost immediately after dosing the reagents), heterogenous materials can be obtained in some cases. When using laboratory mixers, precipitation conditions are not fully controlled, and, locally, slightly different materials may be formed, which is reflected in local differences in the sorbent composition. Therefore, for further optimization, a special reaction system should be designed. Zr-ATMP 7 is the most heterogeneous material and Zr-ATMP 1 and Zr-ATMP 5 are moderately heterogeneous, while the rest of the materials are relatively the most homogeneous materials in this group. Upon comparing the range/mean ratio for Zr-ATMP sorbents with previously published results for Zr(IV), Ce(IV), and Ti(IV) phosphates [33], it should be noted that for the majority of materials, the range/mean ratios are similar and within the range of 5–50%. Only for previously studied crystalline cerium phosphates (CeP1, CeP2, CeP3) [33] was the range/mean ratio lower (1–20%).

All SEM-EDS measurements were carried out for sorbents applied on a conductive carbon tape (typically used in SEM-EDS imaging), so some of the EDS results may be distorted by the analytical signal from the tape. Therefore, SEM-EDS analysis was also recorded for pure carbon tape to estimate how big such interference might be. It turned out that the EDS signal from pure tape showed the presence of not only carbon (ca. 64 atomic%) but also oxygen (ca. 31 atomic%) and nitrogen (ca. 5 atomic%). Other signals were below 0.1 atomic%.

The carbon, oxygen, and nitrogen contents are presented in Table 2, but, due to the interference of the analytical signal by the signal from carbon conductive tape, these results should be interpreted with caution. If the thickness of the analyzed sample was low, the analytical signal from the sample may be “diluted” by the signal from the tape. Because the carbon conductive tape used for SEM-EDS analysis did not contain P and Zr, the P:Zr(IV) molar ratio is not affected by the presence of an analytical signal from the tape in the recorded measurements, and it can therefore be safely interpreted. The P:Zr(IV) molar ratio was higher than one, even if the P:Zr molar ratio in the reaction mixture was lower than one. It is likely that only part of the zirconium ions present in the solution was precipitated in the case of Zr-ATMP 1, which is consistent with the reported yield (45%). It should be mentioned that the P:Zr(IV) molar ratio in the reaction mixture affects the precipitation efficiency. Moreover, it should be mentioned that the yields obtained in the case of Zr-ATMP 1, Zr-ATMP 2, and Zr-ATMP 3 were significantly lower than in the case of the others. Therefore, a high P:Zr(IV) molar ratio should be applied for efficient preparation of Zr-ATMP sorbents. Only at a P:Zr(IV) molar ratio equal to or greater than five does complete precipitation of zirconium ions from the solution occur. The concentration of zirconium ions in the solution in such cases is below 1 mg/L.

The P:Zr(IV) molar ratio usually increases with the increase in the P:Zr(IV) molar ratio in the reaction mixture. The typical range of the P:Zr(IV) molar ratio for materials synthesized at a P:Zr molar ratio in the reaction mixture higher than one is in the range of 2.4 to 5.2. The exception is the Zr-ATMP 8 sample with a P:Zr(IV) molar ratio in the range of 7.0–7.5. A P:Zr(IV) molar ratio higher than two indicates that the material contains free phosphonate (-PO_3_^2−^) and hydrogenphosphonate (-PO_3_H^−^) groups with ion exchange capacity.

Carbon content results are the least reliable because of possible overestimation of the result by the signal coming from the carbon conductive tape. However, the measured results can be used for correction calculations for other elements. Assuming a single source of carbon (from methylene groups of ATMP) in the prepared materials, the expected C:P molar ratio should be constant and equal to one. The measured C:P ratio allowed for a rough estimate of what percentage of the EDS signal comes from the sample and what percentage comes from the carbon conductive tape. It seems that up to 21% of the recorded EDS signal can come from the carbon tape (typically, about 15%). Furthermore, due to the morphology of the sample and grain sizes, additional errors are possible [53]. For this reason, CHN analysis results were added to discuss the trends observed in the EDS results for light elements.

The N content determined through SEM-EDS may also be affected by an error. The expected N:P molar ratio in all samples should be constant and equal to 0.33 (because of the structure of ATMP), but it is not in the analyzed samples. The observed N:P molar ratio is significantly higher than 0.33 in almost all cases, with the exception of Zr-ATMP 7. The N:P molar ratio usually ranges from 0.6 to 1.4, with average values ranging from 0.7 to 1.3. There is no clear trend related to the P:Zr(IV) molar ratio in the reaction mixture. The N:P molar ratio may be overestimated due to the presence of nitrogen in the carbon conductive tape, but, considering that a maximum of 21% of the signal can come from the carbon tape, the maximum correction for the N:P molar ratio is 0.21. Thus, even taking into account the possible maximum deviation, the recorded N:P molar ratio should be lower than 0.54. However, for almost all samples, the N:P molar ratio is higher. Such results suggest the presence of another source of nitrogen atoms in the material than ATMP. This hypothesis was verified based on the CHN results. Most likely is the presence of nitrate ions in the structure of the material (the source of zirconium ions in the synthesis was zirconium oxynitrate).

The O:P molar ratio can also be distorted due to the high oxygen content in the carbon conductive tape. The O:P molar ratio in the ATMP molecule is equal to three, so if the material contains only ATMP-bound zirconium ions, the O:P molar ratio should be also equal to three. In all analyzed cases, the O:P molar ratio is much higher than three, and it ranges from 3.33 to 11.17. The calculated correction for the O:P molar ratio is not greater than 1.3, so if the material contains only ATMP units, the measured O:P molar ratio should not be higher than 4.3, but, for almost all materials, the O:P molar ratio is significantly higher. The highest O:P molar ratio value was observed for Zr-ATMP 1, and this may be related to the lowest P:Zr molar ratio in the reaction mixture. It is likely that in this case, the material contains many zirconium hydroxide moieties. The high O:P molar ratio may also be associated with the presence of nitrite ions (high O:P molar ratio values correspond well to high N:P molar ratios), hydration water, and oxygen contributed by zirconyl ions (ZrO^2+^). It is important to note that zirconium nitrate, even at moderately acidic pH, slowly hydrolyzes, so the precipitation of zirconium aminotris(methylenephosphonate) may be affected by the hydrolysis process. The partial product of hydrolysis is zirconyl ions (ZrO^2+^).

In order to further clarify the composition of the sorbents, the CHN analysis and the determination of the residue on ignition were performed. The results are presented in Table 4. Firstly, it should be mentioned that the sum of the residue after calcination and C, H, and N content is not equal to 100% for all samples. The differences range from 10.4 to 49.5 and cannot be explained by the uncertainty of the analyses. Such large differences are related to the mode of CHN analysis. During combustion, water evaporates from the sample, and ATMP moieties are burned to CO_2_, NO_x_, and H_2_O. Nitrate ions (if present in the sample) also decompose to NO_x_. Phosphorus from ATMP is oxidated to P(V), and part of it in the form of P_2_O_5_ will evaporate (the boiling point of P_2_O_5_ is about 360 °C), while the rest of the P(V) will form zirconium pyrophosphate, which is stable to at least 1000 °C [54]. The elemental analysis was carried out in CHN mode, so evaporated P_2_O_5_ is not measured. Therefore, the difference between the sum of C, H, and N and the residue on ignition is associated with the phosphorus pentoxide evaporated from the sample.

Looking at the values of the residue on ignition, it can be seen that the results form three significantly different groups: Zr-ATMP 1—Zr-ATMP 3 with ROI = 74–78%, Zr-ATMP 4—Zr-ATMP 7 with ROI = 54–60%, and Zr-ATMP 8 with ROI = 33%. The ROI results roughly correspond to the P:Zr(IV) molar ratios measured through EDS (the higher the P:Zr(IV), the lower ROI). Moreover, the ROI results also correspond well to differences associated with evaporated P_2_O_5_ discussed above; for Zr-ATMP 1—Zr-ATMP 3, the difference is 10.4–13.2, for Zr-ATMP 4—Zr-ATMP 7, the difference is 25.5–30.6, and for Zr-ATMP 8, the difference is 49.5%.

To sum up, this is most likely related to the different stoichiometries of Zr-ATMP complexes in the structure of the material. It is possible that species with stoichiometries (Zr:ATMP) of 2:1, 1:1, and 1:2 are formed during precipitation at different molar ratios of zirconium reagent to ATMP. Moreover, as proposed based on the EDS analysis, some sorbents can contain water molecules, nitrate ions, as well as zirconium oxide (or hydroxide) moieties in the structure.

For better understanding, the molar ratios of H:C and N:C were calculated. Assuming that carbon comes only from the ATMP ligand, the N:C molar ratio shows that for the first five materials, it is higher than expected (for ATMP N:C = 0.33). Therefore, most likely, the sorbents from Zr-ATMP 1 to Zr-ATMP 5 contain some nitrate in the structure (about one nitrate ion per three ATMP molecules), while the samples from Zr-ATMP 6 to Zr-ATMP 8 contain nitrogen only from the ATMP ligands.

The H:C molar ratio for the first two sorbents is significantly higher (5.2–5.7) than for the others (3.9–4.6). The sorbents from Zr-ATMP 3 to Zr-ATMP 8, except for Zr-ATMP 6, exhibit a very similar H:C molar ratio (3.9–4.2), which is close to the theoretical H:C molar ratio for the ATMP ligand (equal to 4) and for the mono- and di-deprotonated forms of ATMP (3.33 and 3.67). Nevertheless, these samples contain at least 1.5 water molecules per ATMP unit (assuming that ATMP is doubly deprotonated). The outlier H:C molar ratio for Zr-ATMP 6 is probably associated with the greater amount of water in the structure of the material. Also, in this case, the results roughly correspond (with some exceptions) to O:P molar ratios from the EDS analysis (the higher the H:C molar ratio from the CHN analysis, the higher the O:P molar ratio from the EDS analysis). Therefore, in the case of Zr-ATMP 1 and Zr-ATMP 2, the higher H:C molar ratio can be explained by the greater amount of water in the structure or the presence of zirconium hydroxide moieties.

**Figure 3:**
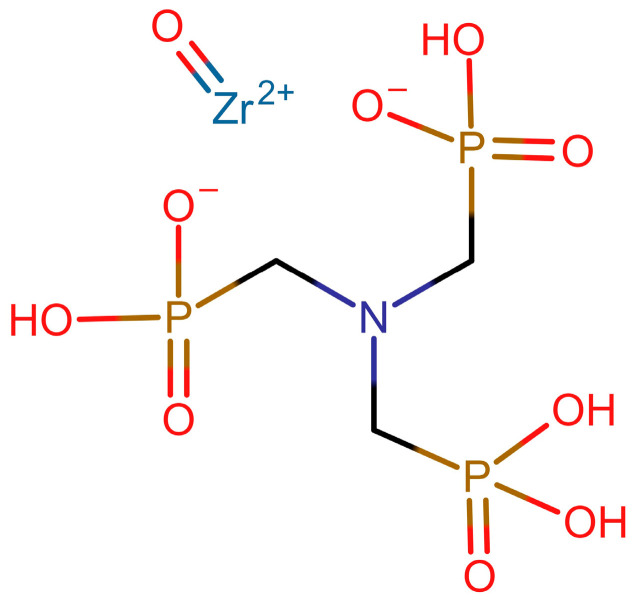
Structure of the zirconyl ions–ATMP 1:1 complex.

Taking into account the above considerations, the composition of the Zr-ATMP 7 sample is close to the theoretical composition of the material with stoichiometry of the 2:1 Zr:ATMP complex described by the formula Zr(ATMP)_2_ ([C] = 10.2 wt%; [H] = 3.2 wt%; [N] = 4.0 wt%; ROI = 37.7%). Samples from Zr-ATMP 4 to Zr-ATMP 7 most likely have stoichiometry closer to the 1:1 Zr:ATMP complex (Figure 3) ([C] = 8.9 wt%; [H] = 2.5 wt%; [N] = 3.5 wt%; ROI = 66%) but are contaminated with additional water and nitrate molecules. The materials probably have stoichiometry between 1:1 and 1:2 Zr:ATMP complexes. The stoichiometry of the samples from Zr-ATMP 1 to Zr-ATMP 3 is closer to the 2:1 Zr:ATMP complex ([C] = 7.1 wt%; [H] = 1.6 wt%; [N] = 2.8 wt%; ROI = 76%), but they are contaminated with additional water and nitrate molecules, as well as zirconium hydroxide.

**Figure 4:**
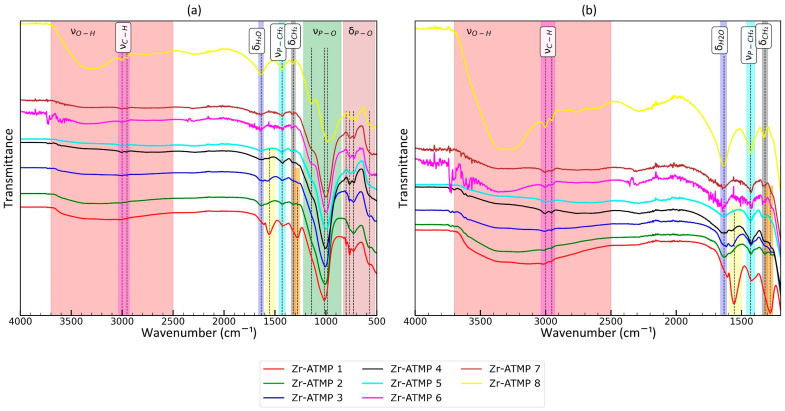
ATR-FTIR spectra of the prepared Zr-ATMP materials: (**a**) full spectrum and (**b**) spectrum fragment in the range of 1200–4000 cm^−1^.

The FTIR spectra were analyzed to further elucidate the structure. The recorded spectra for the prepared materials are presented in Figure 4. The main bands were marked, and their assignment to functional groups is also included in Figure 4 for clearer discussion.

All spectra contain bands associated with C-H stretching vibrations (methylene groups of ATMP moieties) with two maxima at 2953 and 3002 cm^−1^. The intensity of these bands is dependent on the P:Zr(IV) molar ratio used (they are less intense for Zr-ATMP 1 and most intense for Zr-ATMP 8). The frequency of ν_C-H_ is the same in all cases. For all samples, moderately intense, broad, and complex bands in the hydroxyl group (O-H) stretching region (2500–3700 cm^−1^) are observed, but the relative intensity of the components in these bands varies from sample to sample. The most exceptionally intense band was recorded for Zr-ATMP 8, and it is probably associated with the high concentration of bound water in the material structure. This conclusion is also supported by the observation of the exceptionally intense δ_H2O_ band at 1635 cm^−1^. Although deconvolution is necessary for a detailed analysis of the superimposed O-H bands, a preliminary inspection of the raw spectra already reveals that the composition of the bands is highly dependent on the synthesis conditions and critical for sorption properties. The frequency of the δ_H2O_ band (maximum at 1635 cm^−1^) is also the same for all samples of Zr-ATMP sorbents. A comparison of the maximum position of the δ_H2O_ band with previously studied metal(IV) phosphates [33] shows that water must interact with the sorbent matrix, as the position of the band is slightly modified (1625 cm^−1^ for Zr-ATMP sorbents vs. 1640 cm^−1^ for titanium(IV) phosphates vs. 1625 cm^−1^ for zirconium(IV) phosphates vs. 1620 cm^−1^ for cerium(IV) phosphates). Zr-ATMP 1 has a different band arrangement in the 1200–1700 cm^−1^ range. Two additional bands were observed at 1555 cm^−1^ and 1280 cm^−1^. These bands may be associated with the presence of nitrate ions (which would correspond well to the exceptionally high N:P atomic ratio) or surface carbonate groups, which are typically observed in Zr(OH)_4_ exposed to air [55]. It should be noted that both explanations are tentative. Because Zr-ATMP 1 exhibited poor sorption properties, this issue was not explored further. The band with a maximum at 1428 cm^−1^ was present in all cases. The intensity for Zr-ATMP 1 to Zr-ATMP 7 was similar, while for Zr-ATMP 8 it was higher. According to the literature, the band is associated with P-CH_2_ stretching vibrations [36]. Also, for almost all samples, a weak band at 1320 cm^−1^ is visible, which is related to CH_2_ twisting [36]. In the range characteristic of the stretching vibrations associated with phosphonate groups (850–1220 cm^−1^), significant differences between sorbents are observed. It can be assumed that the band consists of at least three components (with maxima at 1142, 935, and 985–1016 cm^−1^). The different intensities of these components for individual materials probably reflect differences in the composition of the surface groups. It is worth noting that the relative intensity of the component at 1142 cm^−1^ increases with the increasing P:Zr(IV) molar ratio in the reaction mixture. The main peak in this band is shifted to lower wavenumbers as the P:Zr(IV) molar ratio in the reaction mixture increases (from 1016 to 985 cm^−1^). A component with a maximum at 935 cm^−1^ is present only in Zr-ATMP 7. The complex structure of the δ_P-O_ band (520–830 cm^−1^) also evolves with changes in the P:Zr(IV) molar ratio in the reaction mixture.

**Figure 5:**
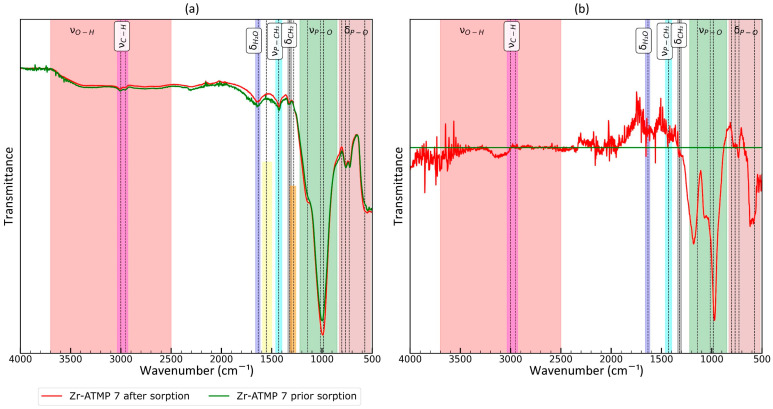
ATR-FTIR spectra of Zr-ATMP 7 prior to and after sorption: (**a**) full spectrum and (**b**) differential spectrum (“after”–“prior”).

To better understand the mechanism of sorption, ATR-FTIR spectra were recorded for the best sorbent (Zr-ATMP 7) before and after sorption (Figure 5). The changes are rather small, so a differential spectrum is also presented. The main differences are observed in the ν_P-O_ region—the intensity of the two components (maxima at 1175 and 971 cm^−1^ for differential spectrum) increases for the sample after sorption. Moderately intense changes are also observed in the δ_P-O_ region (the most intense change is at 600 cm^−1^).

The changes in other regions are small and noisy. It is likely that the amount of water in the sorbent structure has also changed (please see changes at 1640 cm^−1^). The changes in a large part of the ν_O-H_ region are negligible. The exception is a slight increase in absorption in the range from 3005 to 3250 cm^−1^, so only this part of the ν_O-H_ region is probably associated with ion exchange active O-H groups. The results presented here prove that phosphonate groups are involved in sorption. It is important to note that the concentration of copper in the tested solution (Solution S4) was low (20 mg/L), so only a part of the active groups of the sorbent participated in the process, and therefore the changes in the spectrum were expected to be rather low. The presented results also show that FTIR spectroscopy is an effective technique for monitoring the sorption process.

**Figure 6:**
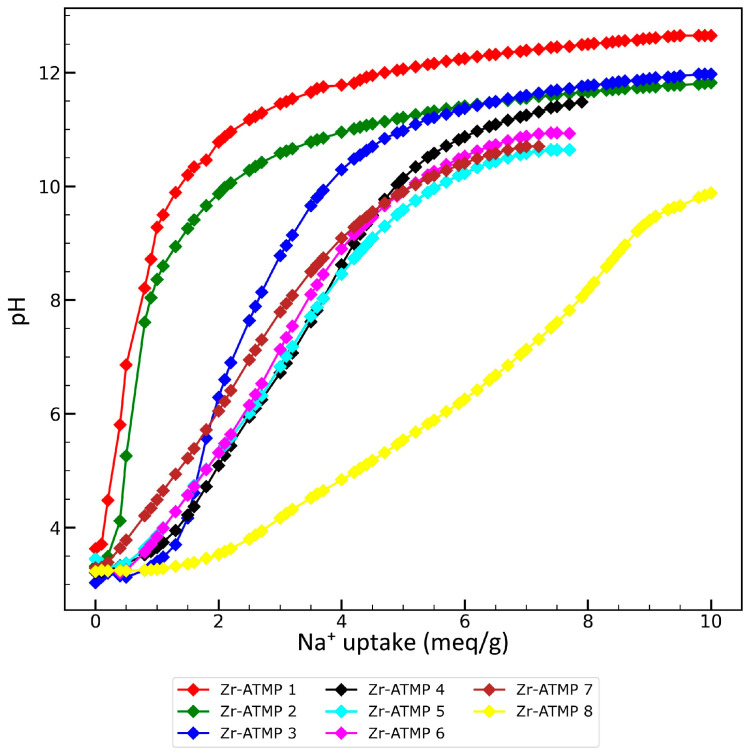
Titration curves of Zr-ATMP sorbents.

The total ion exchange capacity was determined by titrating a suspension of the sorbent in deionized water using 0.1 M NaOH. The results are shown in Figure 6. Almost all of the tested sorbents are not stable in an alkaline environment and start to dissolve at a pH around nine. Because ATMP itself can buffer a homogenous mixture, it is difficult to determine the total ion capacity of the material. Even after complete dissolution of the materials (typically at pH 11–12), the pH increased only slowly after adding subsequent doses of NaOH. Therefore, titrations were stopped when the mixture became transparent. In the case of Zr-ATMP 1, Zr-ATMP 2, and Zr-ATMP 3, the mixtures were not transparent even after the addition of 10 meq/g of NaOH. It is likely that zirconium hydroxide was formed.

All titration curves show a profile that is characteristic of amorphous materials [44,45,46,47] and devoid of clearly separated steps (S-shaped curves) associated with the neutralization of individual functional groups with different acid–base properties (different pK_a_). The ion exchange capacity increases significantly with the P:Zr(IV) molar ratio. Na^+^ uptake is in the range of 2.7–7.9 meq/g for materials synthesized at a P:Zr(IV) molar ratio higher than one at pH equal to eight. These values are higher than those of Na^+^ uptake at pH eight for previously tested amorphous zirconium, cerium, and titanium phosphates (4.5–7 meq/g for Ti(IV) phosphates, 1.5–5 meq/g for Zr(IV) phosphates, and 1.5–3.4 meq/g for Ce(IV) phosphates) [33], so the obtained materials should also exhibit better sorption properties than phosphates.

**Figure 7:**
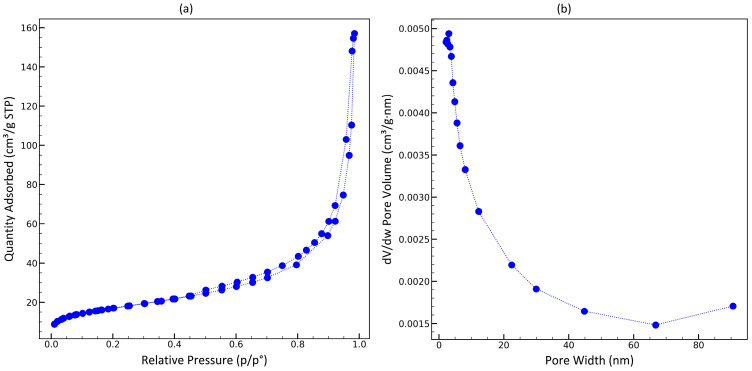
Porosimetry results. (**a**) Nitrogen adsorption–desorption isotherm of Zr-ATMP 7 and (**b**) corresponding pore size distribution curve.

Nitrogen porosimetry was performed for the most promising sorbent, Zr-ATMP 7. As seen in Figure 7, it is a type II isotherm (according to the IUPAC classification) with very small hysteresis. Such results suggest that the material is macroporous. The pore size distribution curve is similar to that reported in the literature [36]. The BET surface area of the sorbent is equal to 62 m^2^/g, and the total volume of pores is equal to 0.23 cm^3^/g. According to the literature, the surface area of the synthesized Zr-ATMP materials decreases significantly with the increase in the P:Zr(IV) molar ratio used during synthesis, e.g., sorbents obtained through the hydrothermal method at a molar ratio of P:Zr(IV) ≥ 3 [36,37] have a surface area of less than 10 m^2^/g. The presented method of precipitation in an aqueous solution under mild conditions allows for obtaining materials with a higher specific surface area at high P:Zr(IV) molar ratios. The higher surface area should have a positive effect on the sorption kinetics. The obtained result corresponds well with the SEM imaging results.

### 3.2. Sorption Properties of Zr-ATMP Materials and Selected Reference Resins in Pure Copper Ions Solution—Study of Sorption Isotherms and Kinetics

The first stage of the sorption tests involved determining the parameters of copper capture by Zr-ATMP sorbents under optimal conditions; therefore, the sorption isotherms and kinetics for pure copper sulphate solutions were examined. For comparative purposes, two commercially available resins (Puromet^TM^ MTS9300, AmberSep^TM^ M4195) showing high affinity for copper were also tested.

**Figure 8:**
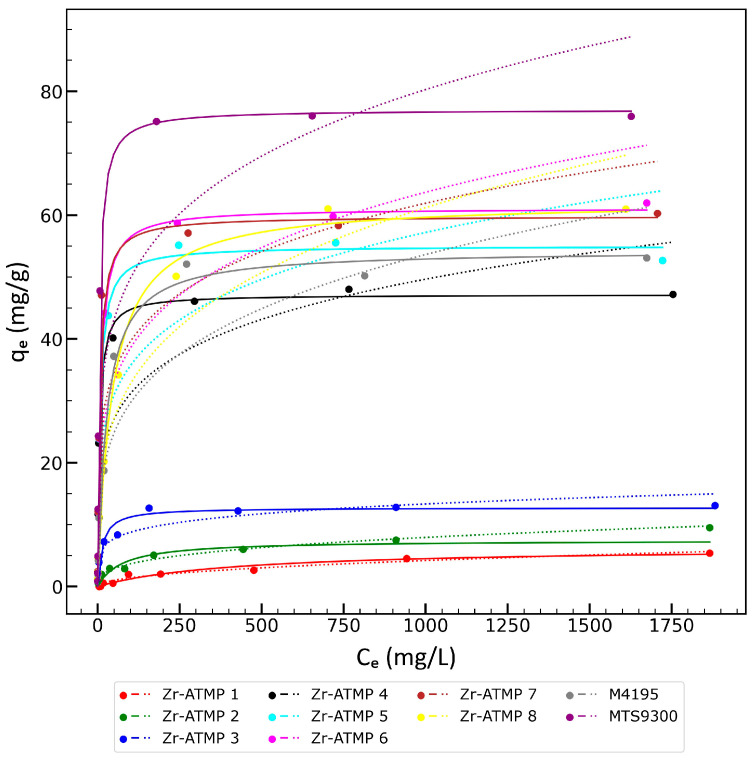
The sorption isotherms for Zr-ATMP sorbents and two commercially available resins. The best-fitted Langmuir model is marked with a solid line, while the Freundlich model is marked with a dotted line.

The results are presented in Figure 8 and Table 5. Materials synthesized at a low P:Zr molar ratio (0.5–2) exhibit weak sorption properties, which is consistent with previously published data for zirconium(IV), titanium(IV), and cerium(IV) phosphates [33]. Materials prepared at higher P:Zr molar ratios (>2) show significantly better sorption properties—the maximum copper sorption capacity ranges from ca. 47 to ca. 62 mg/g. The effect of the P:Zr(IV) molar ratio on the sorption properties of Zr-ATMP materials is similar to the previously observed trends in lanthanides sorption on Zr-ATMP materials synthesized under hydrothermal conditions [36,37], but the maximum sorption capacities achieved were lower than those reported here for copper (despite the fact that lanthanides have higher molar masses). AmberSep^TM^ M4195 and Puromet™ MTS9300 under the same conditions achieved a maximum copper capacity close to 54 mg/g and ca. 77 mg/g, respectively. Thus, the best Zr-ATMP material exhibits higher copper capacity than AmberSep^TM^ M4195 resin and a lower capacity than Puromet™ MTS9300.

The analysis of the fitted Langmuir and Freundlich sorption isotherm models shows that the quality of fit for materials synthesized at low P:Zr molar ratios in the reaction mixture (0.5–2) is lower for the Langmuir model and higher for the Freundlich model, while with the increase in the P:Zr(IV) molar ratio the determination coefficient increases for the Langmuir model and significantly decreases for the Freundlich model. It should be noted that the Freundlich model can be used only for concentrations significantly lower than the concentration at which the sorbent is saturated. Therefore, only the parameters for the Langmuir model are discussed.

**Figure 9:**
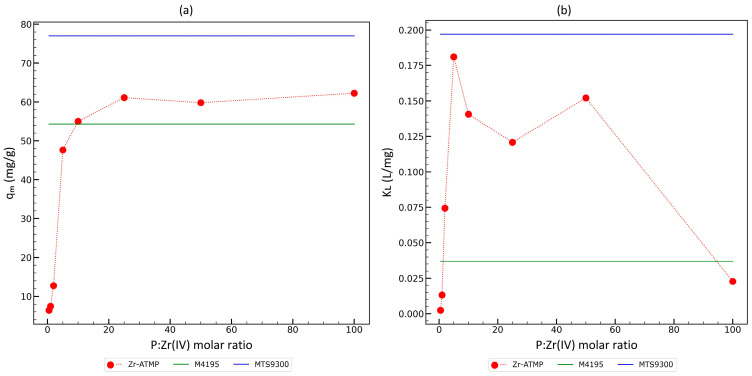
Correlation between (**a**) *q_m_* and P:Zr(IV) molar ratio in the reaction mixture and (**b**) *K_L_* and P:Zr(IV) molar ratio in the reaction mixture.

The correlation between the maximum copper capacity and the Langmuir adsorption constant with the P:Zr(IV) molar ratio in the reaction mixture was also analyzed, and the results were compared with the isotherm parameters for the tested resins (Figure 9). As it can be seen, the prepared materials exhibit a similar or slightly lower maximum copper capacity compared to resins, while the Langmuir adsorption constant for almost all Zr-ATMP materials is significantly higher than the *K_L_* for AmberSep^TM^ M4195 resin and slightly lower than the *K_L_* for Puromet™ MTS9300. Zr-ATMP materials exhibit high affinity to copper ions at low Cu^2+^ concentrations. The presented correlations show that the highest Langmuir constant was achieved for the materials synthesized at a P:Zr(IV) molar ratio in the reaction mixture in the range of 5–50, and further increasing the P:Zr(IV) molar ratio leads to a decrease in the *K_L_* values. The optimal P:Zr(IV) molar ratio in the reaction mixture for achieving the highest copper capacity is 50. Considering *q_m_* and *K_L_*, the optimal P:Zr(IV) molar ratio is in the range of 10 to 50. The optimal P:Zr(IV) molar ratio in the reaction mixture is 50.

The second issue investigated in pure copper sulphate solution was the study of sorption kinetics (Table 6). The adsorption rate of copper ions from pure Cu^2+^ solution is extremely high for Zr-ATMP materials synthesized at a P:Zr molar ratio in the reaction mixture ranging from 5 to 100. It took no more than 3 min to achieve 90% of its equilibrium capacity for solutions containing 5–50 mg/L of Cu^2+^. Materials prepared at lower P:Zr molar ratios exhibit sluggish adsorption kinetics, so such materials not only have poor copper capacity but also the reaction rate with adsorption centers is unfavorable. For the resin tested, it took more than 1 h in the case of AmberSep^TM^ M4195 and about 30 min in the case of Puromet™ MTS9300 to reach 90% of its equilibrium capacity. It should be emphasized that a direct comparison of kinetics for the material in powder form (Zr-ATMP sorbents) and in the form of granules (resins) may be inaccurate, as the powder has a higher surface area and, consequently, lower mass transfer resistance. Nevertheless, the results obtained for Zr-ATMP sorbents are attractive and can indicate advantages over commercially available resins.

### 3.3. Sorption Properties of Zr-ATMP Materials and Selected Reference Resins in Solutions of Increasing Complexity

To assess the affinity of Zr-ATMP sorbents for copper ions, distribution coefficients were determined in four solutions differing in composition, i.e., containing an increasing amount of competitive ions, following the methodology described in our previous study [33]. Tests were performed for two best sorbents: Zr-ATMP 6 and Zr-ATMP 7. The results are presented in Table 7 and compared with previously published data for commercially available resins (for a clearer discussion). Experiments were conducted at a pH of about two.

The *log K_d_* values for selected Zr-ATMP materials determined in pure copper solution were higher than the *log K_d_* values for almost all resins tested in the previous study [33] and comparable to the *log K_d_* value for general purpose cation exchange resin AmberLite^TM^ HPR1200 H. Zr-ATMP 6 and Zr-ATMP 7 also exhibit *log K_d_* values higher than almost all previously tested phosphate materials, with the exception of ZrP5 and CeP3 (please see Table 8 in [33]).

Unlike previously tested phosphates, the addition of 1000 mg/L of sodium and potassium ions to the solution containing copper ions does not reduce the *log K_d_* values for zirconium phosphonate sorbents. This is a significant advantage of these materials. Moreover, in the presence of Na^+^ and K^+^ ions, the *log K_d_* values for Zr-ATMP materials are higher than for all tested resins. Therefore, the obtained amorphous zirconium aminotris(methylenephosphonate) materials can probably be used to capture copper ions from brackish waters.

Only the introduction of calcium (20 mg/L) and magnesium (200 mg/L) ions reduces the *log K_d_* values by one, but Zr-ATMP sorbents still exhibit higher values than all previously tested phosphates, as well as almost all tested resins. Only the *log K_d_* value for AmberSep^TM^ M4195 is comparable to the *log K_d_* values for Zr-ATMP 6 and Zr-ATMP 7, while the *log K_d_* value for Puromet™ MTS9300 is higher by 0.6 [33].

Transition metal salts (Cd, Ni, Zn, Fe, Mn) added to Solution 3 in quantities similar to copper slightly reduce the *log K_d_* values of selected Zr-ATMP sorbents. However, in this complex solution, the *log K_d_* values for Zr-ATMP 6 and Zr-ATMP 7 are also significantly higher than the *log K_d_* values for AmberLite™ HPR1200H, Puromet™ MTS9100, Puromet™ MTS9500, LEWATIT^®^ MonoPlus TP260, and Dianion CR20, comparable to AmberSep^TM^ M4195, and lower than Puromet™ MTS9300 [33].

To sum up, the best Zr-ATMP sorbents show significantly better affinity for copper ions in a complex solution than previously tested phosphates, and, in some cases, they can compete with state-of-the-art commercially available materials.

The selectivity of copper uptake compared to other heavy metals was also analyzed. The separation factors are presented in Table 8. The selected Zr-ATMP materials exhibit a high separation factor only for the separation of Cu^2+^ from Ni^2+^, while rather low separation factor values were found for separation from Cd^2+^, Mn^2+^, Zn^2+^, and Fe^3+^. Therefore, Zr-ATMP materials cannot be considered copper-selective sorbents. However, in many cases, it is desirable to remove several heavy metals simultaneously, so the low separation factor values for heavy metals can be considered an additional advantage. Here, the efficient sorption of Fe^3+^ should be mentioned. It is known that phosphonate sorbents preferentially interact with metals at higher oxidation levels (+3 and +4), such as lanthanides, uranium, and transuranium elements [36,39,43].

The production of high-affinity and high-selectivity adsorption materials is a difficult task, and, even in the case of state-of-the-art commercially available materials designed for copper removal, like AmberSep^TM^ M4195, some separation factors are low and even lower than those of Zr-ATMP materials (*SF_Cu/Cd_* and *SF_Cu/Ni_*). Only the Puromet™ MTS9300 resin exhibits both high affinity and high selectivity under the tested conditions.

It is also interesting to note that the best Zr-ATMP materials exhibit high separation factors for Ni^2+^, while some phosphate materials [33] exhibit high separation factors for Mn^2+^. Such observations suggest that the synthesis of phosphate–phosphonate composite material can be a good way to tune the affinity and selectivity of the studied amorphous materials.

**Figure 10:**
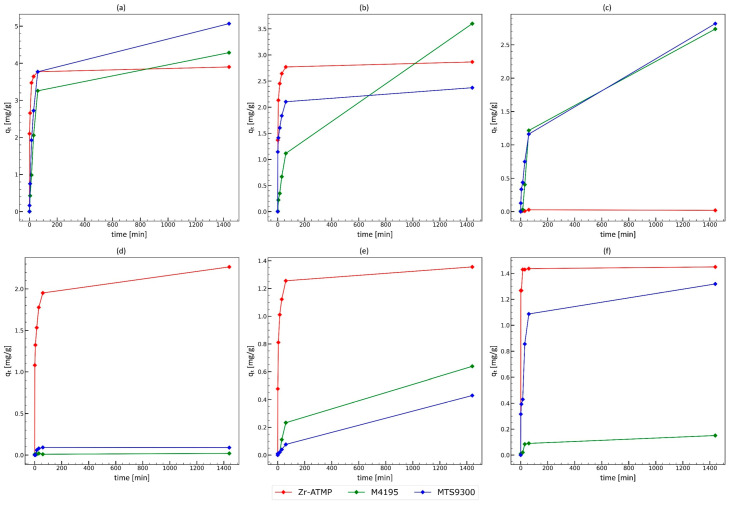
Comparison of the adsorption rate of the tested transition metals from the complex solution for Zr-ATMP 7 material and M4195 and MTS9300 resins. Panel: (**a**) Cu^2+^; (**b**) Cd^2+^; (**c**) Ni^2+^; (**d**) Mn^2+^; (**e**) Zn^2+^; (**f**) Fe^3+^.

Because both selected materials (Zr-ATMP 6 and Zr-ATMP 7) show similar properties, for further studies, only the best one (Zr-ATMP 7) was used. In the next step, the kinetic of transition metal sorption from the complex acid solution was studied and compared with the results for the selected resins. The results are presented in Figure 10. The adsorption rate for Cu^2+^, Cd^2+^, Mn^2+^, Zn^2+^, and Fe^3+^ is significantly higher for Zr-ATMP 7 than for the tested resins, so the prepared material can be used continuously in the process at high flow rates.

**Figure 11:**
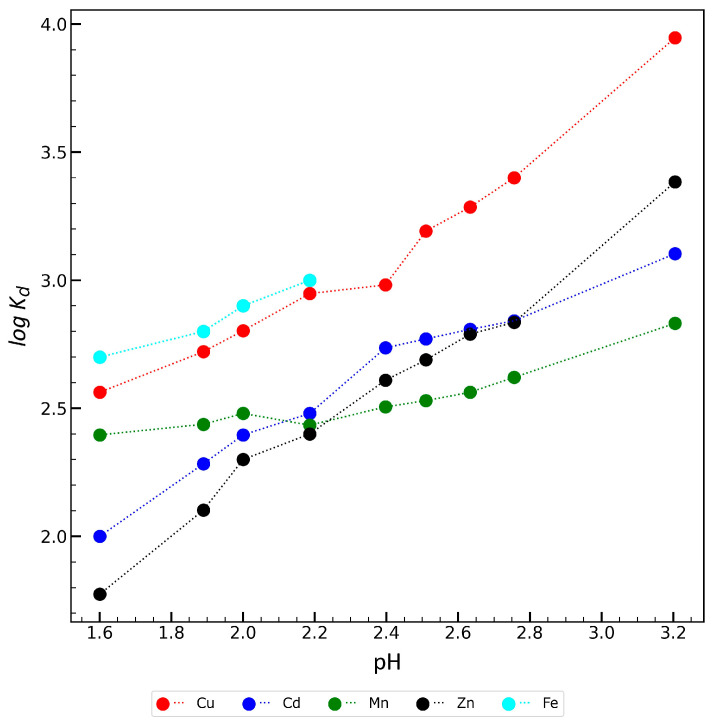
Dependence of pH on *log K_d_* for examined metal ions.

The dependance of *log K_d_* on pH for Zr-ATMP 7 was also examined. The results are presented in Figure 11. Because the *log K_d_* for Ni^2+^ is very low, it is not included in Figure 11. Fe^3+^ ions precipitate significantly at a pH higher than 2.2, so the curve was limited. The affinity for all examined ions decreases with decreasing pH, which is typical for cation exchange materials.

**Figure 12:**
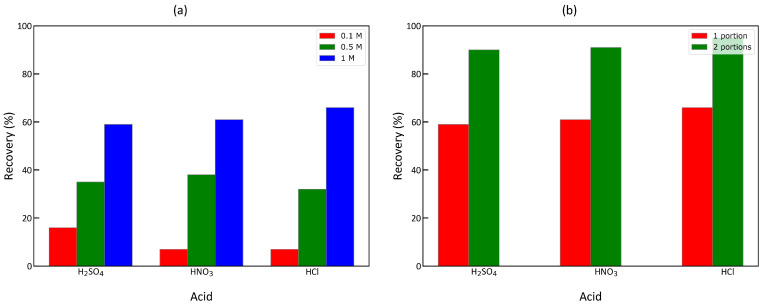
Copper recovery from copper-loaded Zr-ATMP 7 treated with different acids at different concentrations (**a**); cumulative recovery after application of the first and second dose of 1 M solution of selected acids (**b**).

The copper-loaded Zr-ATMP 7 was regenerated using three acids (HCl, H_2_SO_4_, and HNO_3_) at three concentration levels (0.1 M, 0.5 M, and 1 M). The results are presented in Figure 12a. The type of acid used for regeneration did not make much difference. The main driving force was its concentration. Therefore, the desorption process is controlled by the hydrogen ion concentration. The second dose of acid was applied in cases where 1 M acid was used. The cumulative recovery after the second dose of acid solution was higher than 90% (Figure 12b).

Preliminary tests were also carried out to assess the stability of the sorbent. The concentration of leached zirconium was analyzed in the solutions after sorption and regeneration. It turned out that in all tests, the concentration was significantly lower than 1 mg/L.

## 4. Conclusions

Eight fully amorphous Zr(IV) aminotri(methylenephosphonate) sorbents were successfully synthesized under mild and scalable conditions. The prepared materials were characterized with the use of ATR-FTIR, XRD, SEM-EDS, CHN analysis, and pH titration. The performance of the resulting materials was studied both in pure copper solutions and in complex acidic solution (similar to acid wastewater from the copper mining). The results were compared with state-of-the-art commercially available materials designed to capture copper (containing iminodiacetic acid and bispicolylamine as surface groups).

During the study, the effect of the P:Zr(IV) molar ratio in the reaction mixture was analyzed. Materials with the best sorption properties (Zr-ATMP6 and Zr-ATMP 7) were obtained at high P:Zr(IV) molar ratios, i.e., 25:1 (*q_m_* = 61.1 mg/g) and 50:1 (*q_m_* = 59.8 mg/g). Further increasing the P:Zr(IV) molar ratio caused a significant deterioration in the sorption properties of the obtained material. The determined maximum copper capacity for the best Zr-ATMP sorbent is comparable to that for bispicolylamine resin (*q_m_* = 54.3 mg/g) and iminodiacetic resin (*q_m_* = 77 mg/g) determined under the same conditions. The Langmuir constants characterizing the affinity for copper, especially at low levels, are higher for materials obtained at P:Zr(IV) molar ratios of 5–50 (0.12–0.18 L/mg), higher than for bispicolylamine resin (0.037 L/mg), and comparable to iminodiacetic resin (0.197 L/mg). The highest Langmuir constants were achieved for Zr-ATMP 4 (P:Zr(IV) = 5:1).

The analysis of SEM-EDS, CHN, as well as FTIR results indicates that the structure of the resulting materials can be tuned through careful selection of conditions, as zirconium can form a coordination polymer with ATMP with different stoichiometry (e.g., Zr:ATMP complexes 2:1, 1:1, and 1:2) depending on the P:Zr(IV) molar ratio in the reaction mixture and, probably, other parameters.

Almost all Zr-ATMP materials (except for Zr-ATMP 1 prepared at a molar ratio of P:Zr(IV) = 0.5) exhibit excellent sorption kinetics in both pure copper ions and complex acidic solutions. Therefore, the obtained Zr-ATMP sorbents may be used in a continuous process.

For the two best sorbents (Zr-ATMP 6 and Zr-ATMP 7), the affinity for copper ions and sorption selectivity from complex acidic solutions were determined and discussed in relation to previously reported results [33] for commercial resins (with different surface functional groups) dedicated to copper removal. Zr-ATMP 6 and Zr-ATMP 7 exhibit better affinity than AmberLite™ HPR1200H resin (with sulphonic surface functional groups), polyamine-chelating resin Dianion CR20, amidoxime-chelating resin Puromet™ MTS9100, and aminophosphonic resins (LEWATIT^®^ MonoPlus TP260, Puromet™ MTS9500); it is comparable to bis-picolylamine resin AmberSep™ M4195 and worse than iminodiacetic resin Puromet™ MTS9300. Moreover, Zr-ATMP materials exhibit better affinity than all previously reported metal(IV) phosphates [33].

Regeneration studies show that copper-loaded sorbent can be easily regenerated with 1 M acid solutions of HCl, H_2_SO_4_, and HNO_3_. The recovery of copper was up to 95%, so the developed materials can probably be used in a similar manner as chelating resins.

To sum up, our study shows that Zr-ATMP 7 sorbent exhibits promising properties towards copper removal from acidic wastewater, and it can be an interesting alternative to chelating resins in specific applications (e.g., the treatment of acid rock drainage), where low cost and simplicity of production are of great importance. The significant improvement in sorption properties compared to previously tested metal(IV) phosphates [33] indicates that there is room for further optimization in this area. In order to fully assess the usefulness of Zr-ATMP sorbents for copper removal in real applications, additional studies are needed (e.g., investigation of recyclability and long-term stability, experiments in dynamic systems, etc.). Therefore, future research will focus on these aspects.

## Figures and Tables

**Table 1:** Data on the synthesis of zirconium phosphonates.

Sorbent Name	P Source	Zr Source	P:Zr Molar Ratio	Yield (%) ^a^
Zr-ATMP 1	50% ATMP solution	zirconium carbonate basic hydrate reacted with 65% HNO_3_	0.5:1	45
Zr-ATMP 2			1:1	67
Zr-ATMP 3			2:1	87
Zr-ATMP 4			5:1	>99
Zr-ATMP 5			10:1	>99
Zr-ATMP 6			25:1	>99
Zr-ATMP 7			50:1	>99
Zr-ATMP 8			100:1	>99

^a^ yields were calculated based on the results of determining unreacted zirconium ions after sorbent precipitation.

**Table 2:** Composition of Zr-ATMP sorbents determined based on SEM-EDS.

Sorbent Name	Zr(IV) [atomic%]		P [atomic%]		O [atomic%]		N [atomic%]		C [atomic%]	
	Mean	Range ^a^ (rel. Range ^b^)	Mean	Range ^a^ (rel. Range ^b^)	Mean	Range ^a^ (rel. Range ^b^)	Mean	Range ^a^ (rel. Range ^b^)	Mean	Range ^a^ (rel. Range ^b^)
Zr-ATMP 1	3.7	1.1 (30%)	6.4	2.2 (34%)	62.0	12.0 (19%)	8.3	1.3 (16%)	19.6	12.6 (64%)
Zr-ATMP 2	3.7	1.6 (43%)	8.3	2.5 (30%)	60.3	7.5 (12%)	8.1	1.1 (14%)	19.7	3.2 (16%)
Zr-ATMP 3	2.9	0.7 (24%)	7.4	1.6 (22%)	59.4	3.7 (6%)	7.1	0.4 (6%)	23.3	4.1 (18%)
Zr-ATMP 4	2.7	0.9 (33%)	11.4	2.3 (20%)	58.0	8.3 (14%)	7.6	1.0 (13%)	20.3	4.2 (21%)
Zr-ATMP 5	2.9	2.7 (93%)	11.5	4.2 (37%)	56.3	6.5 (12%)	8.3	2.1 (25%)	21.0	2.2 (10%)
Zr-ATMP 6	2.0	0.8 (40%)	7.8	3.0 (38%)	61.3	2.8 (5%)	7.1	0.5 (7%)	21.8	1.3 (6%)
Zr-ATMP 7	4.4	5.1 (116%)	12.3	14 (114%)	49.0	30.9 (63%)	4.8	3.5 (73%)	29.4	52.1 (177%)
Zr-ATMP 8	1.5	0.4 (27%)	10.7	2.1 (20%)	61.7	6.0 (10%)	7.1	0.2 (3%)	19.0	3.8 (20%)

^a^ The difference between maximum and minimum atomic% of an element at the analyzed points on the sorbent surface. ^b^ Relative range equal: 100%∙Range/Mean.

**Table 3:** Molar ratios of P:Zr, N:P, O:P, and C:P calculated based on SEM-EDS.

Sorbent Name	P:Zr(IV) Molar Ratio ^a^	N:P Molar Ratio ^a^	O:P Molar Ratio ^a^	C:P Molar Ratio ^a^
Zr-ATMP 1	1.71 (1.41–1.94)	1.30 (1.15–1.39)	9.68 (8.30–11.17)	3.06 (2.00–5.07)
Zr-ATMP 2	2.24 (2.09–2.52)	0.97 (0.88–1.04)	7.26 (5.68–8.48)	2.37 (2.15–2.74)
Zr-ATMP 3	2.57 (2.42–2.71)	0.96 (0.85–1.12)	8.07 (7.49–9.32)	3.15 (2.63–3.57)
Zr-ATMP 4	4.22 (3.90–4.50)	0.67 (0.64–0.71)	5.09 (4.50–6.34)	1.78 (1.67–1.84)
Zr-ATMP 5	3.97 (3.07–5.21)	0.72 (0.68–0.79)	4.90 (3.69–5.90)	1.83 (1.40–2.22)
Zr-ATMP 6	3.82 (3.74–3.88)	0.91 (0.77–1.04)	7.90 (6.20–9.44)	2.79 (2.20–3.39)
Zr-ATMP 7	2.82 (2.57–3.04)	0.39 (0.33–0.72)	3.98 (3.33–8.00)	2.39 (0.65–17.67)
Zr-ATMP 8	7.32 (7.00–7.54)	0.67 (0.59–0.74)	5.75 (4.87–6.52)	1.78 (1.69–1.82)

^a^ The mean molar ratio calculated based on the results of the SEM-EDS analysis; the range of values from the analysis of individual points is given in brackets.

**Table 4:** Results of CHN analysis and the determination of the residue on ignition at 950 °C.

Sorbent Name	C [wt%]	H [wt%]	N [wt%]	Residue on Ignition [wt%]	N:C Molar Ratio	H:C Molar Ratio
Zr-ATMP 1	5.60	2.45	2.87	78.7	0.44	5.20
Zr-ATMP 2	5.49	2.62	2.77	78.1	0.43	5.67
Zr-ATMP 3	6.72	2.32	3.73	74.0	0.48	4.09
Zr-ATMP 4	7.81	2.75	4.41	55.8	0.48	4.18
Zr-ATMP 5	7.90	2.58	4.35	59.7	0.47	3.88
Zr-ATMP 6	8.23	3.20	3.32	54.7	0.35	4.62
Zr-ATMP 7	8.97	2.99	3.61	54.4	0.34	3.96
Zr-ATMP 8	9.96	3.24	4.35	33.0	0.37	3.86

**Table 5:** Fitting parameters of the Langmuir and Freundlich isotherm models.

Sorbent Name	Langmuir Isotherm Model			Freundlich Isotherm Model		
	*q_m_* (mg/g)	*K_L_* (L/mg)	R^2^	*n*	*K_F_* ((mg/g)·(L/mg)^1/*n*^)	R^2^
Zr-ATMP 1	6.39	0.0024	0.95	2.11	0.158	0.96
Zr-ATMP 2	7.48	0.0132	0.88	2.99	0.789	0.98
Zr-ATMP 3	12.74	0.0744	0.95	5.49	3.788	0.88
Zr-ATMP 4	47.66	0.1811	0.98	4.87	12.18	0.83
Zr-ATMP 5	55.02	0.1406	0.99	4.88	13.9	0.81
Zr-ATMP 6	61.12	0.1208	0.99	4.58	14.09	0.85
Zr-ATMP 7	59.82	0.1521	0.99	5.15	16.19	0.83
Zr-ATMP 8	62.25	0.0228	0.99	3.68	9.359	0.94
M4195	54.33	0.037	0.99	3.89	9.083	0.85
MTS9300	77.01	0.197	0.99	4.81	19.14	0.84

**Table 6:** The time required for a given Zr-ATMP material/resin to reach 90% of its equilibrium sorption capacity in a solution with a defined concentration of copper ions.

Sorbent/Resin Name	Time Required to Reach 90% of the Equilibrium Capacity (min)					
	5 (mg/L)	10 (mg/L)	20 (mg/L)	50 (mg/L)	100 (mg/L)	200 (mg/L)
Zr-ATMP 1	>60	>60	>60	>60	>60	>60
Zr-ATMP 2	>60	>60	>60	>60	>60	>60
Zr-ATMP 3	30	45	60	>60	>60	>60
Zr-ATMP 4	<3	<3	<3	<3	<3	15
Zr-ATMP 5	<3	<3	<3	<3	5	45
Zr-ATMP 6	<3	<3	<3	<3	<3	<3
Zr-ATMP 7	<3	<3	<3	<3	<3	<3
Zr-ATMP 8	<3	<3	<3	<3	<3	<3
M4195	>60	>60	>60	>60	>60	>60
MS9300	30	30	30	30	30	30

**Table 7:** The *log K_d_* values for the most efficient Zr-ATMP materials determined in four solutions differing in complexity of composition.

Sorbent Name	*log K_d_*			
	Solution 1	Solution 2	Solution 3	Solution 4
Zr-ATMP 6	3.9	4.0	2.9	2.7
Zr-ATMP 7	3.8	4.0	2.9	2.8

**Table 8:** The separation factors *SF_Cu/Me_* for copper uptake over the uptake of other heavy metals determined in Solution 4.

Sorbent Name	*SF_Cu/Cd_*	*SF_Cu/Ni_*	*SF_Cu/Mn_*	*SF_Cu/Zn_*	*SF_Cu/Fe_*
Zr-ATMP 6	2.5	330	2.1	3.0	0.9
Zr-ATMP 7	2.6	340	2.0	3.2	0.8

## Data Availability

The original contributions presented in this study are included in the article. Further inquiries can be directed to the corresponding author.

## Abbreviations

The following abbreviations are used in this manuscript:

ATMP	aminotris(methylenephosphonic acid)
Zr-ATMP	zirconium aminotris(methylenephosphonate)
ATR	attenuated total reflection
FTIR	Fourier transform infrared spectroscopy
SEM	scanning electron microscope
EDS	energy dispersive X-ray spectroscopy
XRD	X-ray diffraction
WHO	World Health Organization
IUPAC	International Union of Pure and Applied Chemistry
AAS	atomic absorption spectrometry
BET	Brunauer–Emmett–Teller
BJH	Barrett–Joyner–Halenda
ICP-MS	inductively coupled plasma mass spectrometry
ROI	residue on ignition
